# Supplementary material for: Prevalence and factors associated with Schistosoma mansoni infection among primary school children in Kersa District, Eastern Ethiopia
Source: PeerJ. 2024 Jun 14;12:e17439. doi: 10.7717/peerj.17439 (PMC11182021; doi:10.7717/peerj.17439)
Supplement: Supplemental Information 1 [file peerj-12-17439-s001.docx]

STROBE Statements

|  | Item No |  |
| --- | --- | --- |
| **Title and abstract** | 1 | **Prevalence and Factors Associated with Schistosoma Mansoni Infection among Primary School Children in Kersa District, Eastern Ethiopia** |
|  |  | **Background:** Schistosomiasis is a neglected tropical disease and an important parasite worldwide negatively impacting socio-economic factors. Ethiopia, federal ministry of health targeted the elimination of schistosomiasis infection in school-aged children by 2020. However, *Schistosoma mansoni* still affects approximately 12.3 million school-aged children in Ethiopia. Although the study was conducted in some regions of the country, previous studies were conducted on urban school children and were limited to the burden of infection. Overall, there is lack of information about schistosomiasis in eastern Ethiopia, particularly among school children. Therefore, this study aimed to assess the prevalence and factors associated with *Schistosoma mansoni* infection among primary school children in Kersa district, Eastern Ethiopia.  **Methods**: A cross-sectional study was conducted among 419 school children using systematic random sampling from April 10 to May 09, 2019. The stool samples were collected and examined using the Keto-Katz method. A pretested structured questionnaire was used to collect data from participants. Data were entered using EpiData version 3.1 and analyzed using SPSS version 24. Bivariable and multivariable logistic regression analyses were used to identify factors associated with *Schistosoma mansoni* infection. P-value<0.05 and AOR (95% CI) were used to identify statistically significant associations.  **Results**: The overall prevalence of *S. mansoni* in this study was 19.4% (95% CI, 16, 23). Absence of the latrines in household (AOR=2.35, 95% CI, 1.25-4.38), swimming in the river (AOR=2.82, 95% CI, 1.33-5.88), unprotected water sources (AOR=3.5, 95% CI, 1.72-7.10), irregular shoe wearing habits (AOR=2.81, 95% CI, 1.51-5.23), and water contact during cross of river (AOR=2.192; 95%CI: 1.113, 4.318) were factors independently associated with *S. mansoni* infection.  **Conclusion**: *Schistosoma mansoni* infection remains a public health problem in the study area. Using a latrine in each household, using protected water, wearing shoes regularly, and reducing water contact were necessary to control *Schistosoma mansoni* infection.  **Keywords**: *Schistosoma mansoni,* Infection, kato-katz, School children, Ethiopia. |
| Introduction | | |
| Background/rationale | 2 | Schistosomiasis is one of a neglected tropical infectious disease that affected more than 220 million people worldwide in 2017, of which approximately 90% of them live in Sub-Saharan Africa. According to the WHO definitions, schistosomiasis is an acute and chronic parasitic disease caused by leeches (trematode) of the genus Schistosoma. Human schistosomiasis is caused by trematodes of the genera Schistosoma by the species *Schistosoma mansoni*.  According to the Global Burden of Diseases (GBD) 2016, global disability-adjusted life years (DALYs) are expected to reach 15 million disability-adjusted life years owing to NTDs worldwide. Of these, 3.3 million DALYs were caused by soil-borne helminths, including 1.9 million DALYs attributed to schistosomiasis.  Schistosomiasis is the second most common tropical disease after malaria and infects over 219.9 million people globally, of whom approximately 54.7% are school-age children. They most commonly infect people living in poor communities that lack access to clean water, and adequate sanitation. Intestinal schistosomiasis, caused by S.mansoni manifest as chronic pain accompanied by bloody diarrhoea and intestinal ulcers, while chronic infections lead to liver enlargement, liver fibrosis, portal hypertension, and hematemesis. Schistosomiasis which is associated with a long-term chronic inflammatory response, contributes to anaemia and malnutrition, which in turn can lead to stunted growth, poor academic performance, low work productivity, and persistent poverty. Infection can also increase the risk of malnutrition in children, leading to cognitive impairment and reduced physical function.  Schistosomiasis has been brought under control in many countries but remains a serious public health problem with serious economic consequences in areas where hygiene and control measures are inadequate and the majority of the population lives in poverty. According to the WHO, at least about 75% of school-age children were protected from infection at the end of 2010, yet it was not succeeded.  In Ethiopia, the Federal Ministry of Health (FMoH) has set itself the goal of eliminating schistosomiasis infection in school children through deworming by 2020. However, schistosomiasis remains a major health problem in developing countries, affecting approximately 12.3 million school-age children in Ethiopia. Furthermore, the previous studies were conducted only on urban school children and were limited to the burden of infection.  This study was crucial because the study site was suitable because there was a river between school and residential area of children’s family, also there was irrigational farming for agricultural products around the river, which is suitable for *S. mansoni* intermediate host. An effort has been made to map the epidemiology of *S. mansoni* infections in different geographical region of the country. It is not possible to say that the disease's geographic distribution has been completely mapped out, as there are a recent finding reports new transmission foci that may related to the expansion of water development projects for irrigation and human movement.  Overall, there is lack of information about schistosomiasis in eastern Ethiopia, particularly among school children. Therefore, the aim of this study was to assess the prevalence and factors associated with Schistosoma mansoni infection among primary school children in Kersa district, eastern Ethiopia.  The findings will strengthen existing knowledge and support policymakers to come up with effective *S. mansoni* infection prevention strategies |
| Objectives | 3 | General Objective: To assess the prevalence and factors associated with *schistosoma mansoni* infection among primary school children in Kersa district, East Hararghe zone of Oromia regional state, Eastern Ethiopia from April 10 to 30, 2019.  Specific Objectives   1. To determine the prevalence of *schistosoma mansoni* infection among primary school children in Kersa district, Eastern Ethiopia. 2. To identify factors associated with *schistosoma mansoni* infection among primary school children in Kersa district, Eastern Ethiopia. |
| Methods | | |
| Study design | 4 | Cross-sectional study design was conducted |
| Setting | 5 | A study was conducted in Kersa District, Oromia, Eastern Ethiopia located 493 KM away from Addis Ababa the capital city of Ethiopia, and 33 KM from Harar city. The Kersa district has three climate zones with altitudes ranging from 1,600 to 3,200 meters above sea level. Rain generally falls in two seasons, the main rainy season from June to September and the short rainy season from March to May. The annual rainfall varies between 1,400 to 1,900 mm (*Report***,** Document 2017/2018, Kersa District Agricultural Office). The district is characterized by a warm climate where the annual temperature varies between 18°C and 26°C. The district has 31 primary schools and 27861 students enrolled in 2018/19 (*Kersa District Education Office Report, 2018/19*). The study was conducted among 419 school children from April 10 to May 09, 2019. |
| Participants | 6 | The source population consisted of all children enrolled in grades 1 to 8 of the 31st elementary school in Kersa district during the 2018/2019 school year. The study population was all children attending the selected primary schools. The study included children who attended primary school and had lived in Kersa district for at least six months. Children who had been transferred from a school outside Kersa district within the past six months, who were critically, and had been treated of *S. mansoni* within the past three months were excluded from the study. . Study participants were selected through simple random sampling from each selected primary school by using class rosters as a sampling frame after proportionally allocated to each school. |
| Variables | 7 | **Dependent variables**  - *S. mansoni* infection   **Independent variables**   1. **Socio-demographic characteristics**:  - Sex of respondent, Age of respondent, Grade (educational level of children), fathers’ occupation, Mothers’ occupation, Fathers Educational status, Mothers educational status  1. **Environmental factors**  - Latrine availability at living home, source of water at home, crossing of river/stream on transit to school, proximity of water body to living home - Proximity of water body to school - participating in irrigated farm  1. **School Health (institutional factors)**  - Provision of health education by Health workers at school - Deworming for s. mansoni in last three months - Latrine availability at school - Source of water at school   **D. Personal factors**   - Swimming in contaminated water - Frequency of swimming - Bathing in the rivers/ ponds - Washing of clothes at river - Regular shoes wearing habits - Eating raw vegetable |
| Data sources/ measurement | 8* | A pretested structured questionnaire adapted from a previous study (22-25) was used. The questionnaire includes sociodemographic characteristics, environmental factors, personal factors and *S. mansoni* infection. Factors assessed included sociodemographic, environmental, personal, and other health-related factors. The questionnaire was prepared in English, then translated into the local Afan Oromo language and re-translated to English. Six trained data collectors were deployed to collect data under the supervision of two public health officers. The principal investigator conducted training for the data collectors and supervisors.  **Stool Sample Collection, Processing, and Examination**  Written consent was obtained from the children’s parents/guardians. To ensure the accuracy of the information, the youngsters participating in the study were interviewed in their native language. For students who were unable to answer the questions correctly, guardians were contacted through the school principal.  Students selected for the study were given instructions on how to collect stool samples, as well as how to use an applicator stick, toilet paper, and a clean, labelled plastic container. Immediately upon receipt of stool samples, labelling, quantity, timing, and collection method were reviewed for each sample. Intestinal schistosomiasis eggs were examined in stool samples using a method known as the Kato-Katz technique (26). The Kato-Katz method is the gold standard technique that offers numerous advantages, including: It is performed to increase the likelihood of parasite detection, has high sensitivity, and requires minimal infrastructure. Kato-Katz was used to prepare stool smear on slides for microscopic examination at the Water and Kersa Health Center (27). To determine the intensity of the infection, 42 grams of feces were collected (28, 29).  To ensure the quality of the results, the principal investigator conducted a two-day training session for the data collectors and supervisors on how to conduct the interview and collect stool samples. Completeness of questionnaires was checked and the laboratory tests were carried out by experienced medical laboratory professionals. |
| Bias | 9 | We were used simple random Samling method to reduce selection bias |
| Study size | 10 | The sample size was calculated by Epi-Info version 7.2 using a single population proportion formula for estimating the prevalence of *S. mansoni* infection, and two population proportions formulas for factors associated with *S. mansoni* infection. Therefore, a larger sample size was considered for the study. Accordingly, the sample size for the prevalence of *S. mansoni* infection was calculated using the following assumptions: 45% proportion of S. mansoni infection (21), 95% confidence level, 5% margin of error and 10% non-response rate and accordingly, a minimum of 419 participants required to conduct the study.  Seven primary schools were randomly selected from 31 primary schools in the Kersa district. The sample size was distributed proportionally to each selected primary school based on the number of children enrolled in the school during the 2018/2019 academic year. |
| Quantitative variables | 11 | Quantitative variables in our data were handled by categorizing them into group. For example, age was categorized into three group, 1= “6-9”, 2= “10-14”, and 3= “15-18”. We handled like this. |
| Statistical methods | 12 | After checking for completeness, the collected data were entered using EpiData version 3.1 and analyzed using SPSS version 24. Descriptive statistics such as frequencies, tables and figures were used to present the data. Bivariate and multivariable logistic regression analyses were used to identify factors associated with *S. mansoni* infection. Multicollinearity was checked by using correlation coefficient between independent variables. Adjusted odd ratio (AOR) with 95% CI was applied to determine the association between the dependent and independent variable. The statistical significance was declared at P<0.05. |
|  |  | By running all data on frequency analysis |
| Results | | |
| Participants | 13* | Initially total of 419 were eligible and only 413 primary school children participated in the study with a response rate of 98.6%. |
|  |  | Six were nonrespondents. |
| Descriptive data | 14* | The mean (±SD) age of the participants was 11.33 (±SD 2.72) years ranged from 6 to 18 years old. Majority of participants (240 (58.1%)) were found in the age group of 10-14 years old, followed by 118 (28.6%) in the age group 7- 9 years and 55 (13.3%) 15-18 years old.  Regarding the gender of the participant, about 285 (69 %) were males. Among participants' education levels, most of them were 271 (65.6%) students in grade 1- 4, and 142 (34.4%) were students in grade 5- 8. Two hundred eight three (68.4%) of the participants’ fathers and about 342 (82.6%) of their mothers had no formal education. Of the family occupations, 381 (92%) of the school children’s fathers were farmers, 26 (6.3%) merchants, and 4 (1.0%) government employees. Regarding environmental factors about 243 (58.8 %) participants used water from rivers and streams for drinking and other domestic needs. Nearly half of the participants (46.97%) did not have access to restrooms and instead relied on open fields. About 77 (18.6%) of children had practiced regular hand washing, 183 (44.2%) had washed their hands inconsistently, and 154 (37.2%) had not washed their hands at all after defecation. The majority of children (259, 62.71%) bathe in rivers and streams, while nearly four fifths (77.48%) of schoolchildren wash their clothes in rivers or ponds. One hundred sixty-two (39.2%) youngsters had a habit of swimming. The shoe was worn by just over half (54.24%) of the schoolchildren. One in every three (33.4%) school children’s families were use irrigated agricultural fields as a source of income |
| Outcome data | 15* | The prevalence of S. mansoni infection in this study was 19.4%(80). |
| Main results | 16 | The prevalence of S. mansoni infection was 19.4% (95% CI, 16, 23). The prevalence of S. mansoni by the school among the studied schools ranges from 11.53% in Ejersa Rufa primary school to 36.73% in Beha Biftu Primary School. In binary logistic regression latrine, water source, swimming in rivers or ponds, bathing in rivers or ponds, washing clothes in rivers, family irrigation activities, habits of wearing shoes, and crossing rivers or streams on the way to school were all found to be significantly associated with *S.mansoni* infection. However, in multivariable logistic regression, only the following factors remained significantly associated with *S.mansoni* infection: lack of a toilet at home, river swimming, bathing, habit of wearing shoes, crossing the river to get to school, and sources of water.  The children whose families did not have a latrine at home were 2.34 times more likely infected with *S. mansoni* than those whose family has latrine at home (AOR= 2.34; 95% CI: 1.25, 4.38). In contrast, *S. mansoni* infection was 2.8 times higher in children swimming in the river (AOR= 2.80; 95%CI: 1.33, 5.88) compared to who don't swim at all. The odds of children not wearing the shoes were 2.81 times more likely infected with *S.mansoni* than children with the habits (AOR=2.81; 95% CI: 1.51, 5.23). The children crossed the river to get to the school had higher odd of *S.mansoni* infection than their counterparts (AOR= 2.19, 95% CI: 1.11, 4.32). Ultimately, the proportion of children whose families used rivers as a source of drinking water were more likely to become infected with *S. mansoni* than those who used tap water (AOR= 3.49; 95% CI: 1.72, 7.10) |
| Discussion | | |
| Key results | 18 | According to the current study, the prevalence of S*. mansoni* infection among school children was 19.4% with 95% (CI, 16, 23). Not having a latrine at home, swimming in the river, bathing in the river, shoes wearing habit, crossing the river to get to school, and sources of drinking water were factors statistically associated with S*. mansoni* infection. |
| Limitations | 19 | Limitation of this study was the study only used single stool sample which may not address day-to-day and intra-stool variations of egg that may results in low prevalence. |
| Interpretation | 20 | The present study showed that children using river/stream water for drinking were 3.5 times more likely infected with *S. mansoni* than those who were using tap water. This finding was supported by the studies conducted in Mekelle, Northern Ethiopia (22), and Kisantu, Democratic Republic of Congo (45). This might be due to rivers/streams are usually contaminated by human and animal excreta which result in parasitic transmission when contaminated water were used for drinking or other purpose by school children (46).  However, this finding was not in agreement with the report from Ejaji Town, Central Ethiopia which indicated the source of water for drinking was not significantly associated with *S. mansoni* infection (44).  In addition, school children who had not worn shoes regularly were 2.8 times more likely infected with *S. mansoni* than those who had worn shoes regularly. this finding was similar to the study conducted in Gamo Gofa and South Omo (47), Jiga , Northwest Ethiopia (23), Mana, South West Ethiopia (48) and Zarima, Northwest Ethiopia (49). This might be due to walking bare-foot exposed children to cercariae-infected water when they cross the river to the school which expose them to this infection. This indicates that in addition to lack of education about schistosomiasis water-contaminated transmission, there is a poor information about other soil-transmitted helminths transmission. So, the concerned body like local health planners should educate the community on how to prevent themselves from soil transmitted diseases.  The results of this study showed that the odd of *S. mansoni* infection was 2.2 times higher in school-age children who had physical contact with water while crossing water in a river or stream than in children who did not have physical contact. (AOR=2.2; 95% CI: 1.11, 4.32). This finding was in line with the study conducted in Tigray, Northern Ethiopia (22), Jimma, Southwest Ethiopia (48), and Jiga, Northwest Ethiopia (23). However, this study was not in agreement with the finding from Ejaji Town, Central Ethiopia (44). This might be due to differences in the location of the rivers/streams containing *S. mansoni cercariae* that the children came into contact with when crossing the water, as well as the school's proximity to the river, which allows the children to frequently cross the water and encounter them to bathe and swim together, resulting in direct contact of the intermediate host snail mollusc with the human definitive host and transmitting schistosomiasis (50). Also, differences in factors among the different communities appear to be related with environmental sanitation, water supply and socioeconomic status of households.  This finding reveals that School children who had swum in the river/ponds were 2.8 times higher odds to be infected with *S. mansoni* than those who do not swim in the river/ponds. Similar findings reported from Wolaita, Southern Ethiopia (24), Northwest Ethiopia (25), Zarima Town, Northwest Ethiopia (49) and supported by the reports of a world health organization that, school-aged children swimming in infested water are at higher risk to be infected with *S. mansoni* (51) , (52). This might be due to the whole body’s exposure to cercariae-infected water bodies during swimming that ease transmission (31).  Present study indicated that water contact habit of school children affirmed that swimming and bathing habits in rivers was significantly associated with higher odds to be infected with *S. mansoni*. This finding was in agreement with the studies conducted in Northwest Ethiopia (25), a study from Jimma Zone, South West Ethiopia (48), and study from Ejaji town, Central Ethiopia (44). The main reason for this significant association might be due to proximity of the school to the river bodies occupied with infected snails. |
| Generalisability | 21 | Actually, can be generalized target population, even if its small correctional study it can be generalized to school children as we had randomly selected 7 different school from school found in Kersa district and select participants by using simple random Samling after proportionally allocated to each school. |
| Other information | | |
| Funding | 22 | The study was funded by Haramaya University as part of an MSc study to HA. The funder has no role in the design, execution, analysis, or decision for publication. |
