# Supplementary material for: Prevalence and factors associated with Schistosoma mansoni infection among primary school children in Kersa District, Eastern Ethiopia
Source: PeerJ. 2024 Jun 14;12:e17439. doi: 10.7717/peerj.17439 (PMC11182021; doi:10.7717/peerj.17439)
Supplement: Supplemental Information 3 [file peerj-12-17439-s003.docx]

**Questionnaires for primary school children**

Serial no..........................

Name of school: ..................................................

Grade -------------------------------------

Section -------------------------------

Date of interview: ...................................................

Name of interviewer: .............................................

| **101** | **variable** | codebook  **(Value and Label)** | |
| --- | --- | --- | --- |
| 1 | Identification number | None | |
| 2 | Name of school | 1. “ADELE” 2. “SADOYE” 3. “EJERSA RUFA” 4. WATER 5. “BEHA BIFTU” 6. “BERAKA” 7. “RASA JENETA” | |
| 3 | Grade of respondent | 1=1  2=2  3=3  4=4  5=5  6=6  7=7  8=8 | |
| 4 | Section of respondent | 1=A  2=B  3=C | |
| 5 | Age of respondent | None | |
| 6 | sex of respondent | 1. MALE 2. FEMALE | |
| 7 | What is the religion of respondents? | 1.MUSLIM  2.ORTHODOX  3.PROTESTANT  4.OTHER | |
| 8 | Occupation of your Father? | 1. FARMER 2. MERCHANT 3. EMPLOYEE 4. OTHER | |
| 9 | Occupation of your Mother? | 1. FARMER 2. MERCHANT 3. EMPLOYEE 4. OTHERS | |
| 10 | What is your father's Educational status? | 1. ILLITERATE 2. LITERATE | |
| 11 | What is your mother's educational status? | 1. ILLITERATE 2. LITERATE | |
| 12 | Do you have a latrine at home? | 1. NO 2. YES | |
| 13 | Defecation site at home? | 1. INDOORLATRINE 2. OPENFIELD | |
| 14 | Proximity of your home from Water bodies? | 1. FARAWAY 2. NEAR 3. IDONTKNOW | |
| 15 | Proximity of your school from Water bodies? | 1. FARAWAY 2. NEAR 3. IDONTKNOW | |
| 16 | Where do you get water for drinking at school? | 1. PIPE 2. RIVER | |
| 17 | Where do you get water for drinking at home from? | 1. PIPE 2. RIVER | |
| 18 | Do you pass through water on your way to school? | 1. No 2. Yes | |
| 19 | Have you been engaged in irrigation activities? | 1. No 2. Yes | |
| 20 | Frequency of participation in irrigation | None | |
| 21 | prophylaxisis for schistosomiasis | 1.YES  2. NO | |
| 22 | Provision of health education on Schistosoma at school | 1. No 2. Yes | |
| 23 | Latrine availability at home | 1. No 2. Yes | |
| 24 | Have you been swimming in the river/pond? | 1. No 2. Yes | |
| 25 | How Frequent do you swimming? | 1 Always  2.Sometimes  3. Occasionally | |
| 26 | Do you Bathing in the river? | 1. No 2. Yes | |
| 27 | Have you Washing clothes in rivers? | 1. No 2. Yes | |
| 28 | Have you been washing your hand after defecation? | 1.Yes  2. No | |
| 29 | What frequency you washing hand after defecation? | 1.Regular  2. Irregular | |
| 30 | Have you been eating of raw vegetable? | 1. No 2. Yes | |
| 31 | habits of shoes wearing? | **0.** Always  1. Sometimes | |
|  | Frequency of wearing shoes? | 1. Always  2.Sometimes  3. never | |
| **32** | **age category** | 1.”6-9”  2. “10-14”  3. “15-18” | |
| 33 | Stool sample results | 1. Negative 2. Positive | |
|  | **Checked by supervisor:**  **Name**--------------------------**Signature** --------------------------  Thank you for participating in this interview |  |  |
