# Supplementary material for: Prevalence and factors associated with Schistosoma mansoni infection among primary school children in Kersa District, Eastern Ethiopia
Source: PeerJ. 2024 Jun 14;12:e17439. doi: 10.7717/peerj.17439 (PMC11182021; doi:10.7717/peerj.17439)
Supplement: Supplemental Information 4 [file peerj-12-17439-s004.docx]

**Questionnaires for primary school children**

Serial no..........................

Name of school: ..................................................

Grade -------------------------------------

Section -------------------------------

Date of interview: ...................................................

Name of interviewer: .............................................

| **101** | **DEMOGRAPHIC FACTORS** | **Response** | **Remark** |
| --- | --- | --- | --- |
| 1 | Identification number | -------------- |  |
| 2 | Name of school | -------------- |  |
| 3 | Grade of respondent | -------------- |  |
| 4 | Section of respondent | -------------- |  |
| 5 | Age of respondent | _________ |  |
| 6 | sex of respondent |  |  |
|  | Male |  |  |
|  | Female |  |  |
| 7 | What is the religion of respondents? |  |  |
|  | Muslim |  |  |
|  | Orthodox |  |  |
|  | protestant |  |  |
|  | Others |  |  |
| 8 | Occupation of your Father? |  |  |
|  | Farmer |  |  |
|  | merchant |  |  |
|  | government employee |  |  |
|  | others |  |  |
| 9 | Occupation of your Mother? |  |  |
|  | Farmer |  |  |
|  | Merchant |  |  |
|  | Government employee |  |  |
|  | Others |  |  |
| 10 | What is your father's Educational status? |  |  |
|  | illiterate |  |  |
|  | Read and Write |  |  |
|  | Primary school |  |  |
|  | High school |  |  |
|  | Diploma /degree |  |  |
| 11 | What is your mother's educational status? |  |  |
|  | Illiterate |  |  |
|  | Read and Write |  |  |
|  | Primary school |  |  |
|  | high school |  |  |
|  | Degree/ diploma |  |  |
| **202** | **ENVIRONMENTAL FACTORS** |  |  |
| 12 | Do you have a latrine at home? |  |  |
|  | Yes |  |  |
|  | No |  |  |
| 13 | Defecation site at home? |  |  |
|  | Indoor latrine |  |  |
|  | Open field |  |  |
| 14 | Proximity of your home from Water bodies? |  |  |
|  | Far away (>15 minutes) |  |  |
|  | Near water bodies (<15 minutes) |  |  |
|  | I don’t know |  |  |
| 15 | Proximity of your school from Water bodies? |  |  |
|  | Far away (>15 minutes) |  |  |
|  | Near water bodies (<15 minutes) |  |  |
|  | I don’t know |  |  |
| 16 | Where do you get water for drinking at school? |  |  |
|  | Tap/borehole water |  |  |
|  | River |  |  |
|  | Stream |  |  |
|  | Well |  |  |
| 17 | Where do you get water for drinking at home from? |  |  |
|  | Pipe water |  |  |
|  | River / Stream /pond |  |  |
| 18 | Do you pass through water on your way to school? |  |  |
|  | Yes |  |  |
|  | No |  |  |
| 19 | Have you been engaged in irrigation activities? |  |  |
|  | Yes |  |  |
|  | No |  | If no skip Q.24 |
| 20 | If Ques. 23 yes, how frequent? |  |  |
|  | Always |  |  |
|  | Once per weeks |  |  |
|  | Once per months |  |  |
|  | Some times |  |  |
| **303** | **School Health** |  |  |
| 21 | Deworming for schistosomiasis in the last three months at school? |  |  |
|  | Yes |  |  |
|  | No |  |  |
|  | I don’t know |  |  |
| 22 | Provision of health education on Schistosoma at school |  |  |
|  | Yes |  |  |
|  | No |  |  |
| 23 | Latrine availability at school |  |  |
|  | Yes |  |  |
|  | No |  |  |
| **404** | **PERSONAL FACTORS** |  |  |
| 24 | Have you been swimming in the river/pond? |  |  |
|  | Yes |  |  |
|  | No |  | If no, go to Q.29 |
| 25 | How Frequent do you swimming? |  |  |
|  | Always |  |  |
|  | Some times |  |  |
|  | Occasionally |  |  |
| 26 | Do you Bathing in the river? |  |  |
|  | Yes |  |  |
|  | No |  |  |
| 27 | Have you Washing clothes in rivers? |  |  |
|  | Yes |  |  |
|  | No |  |  |
| 28 | Have you been washing your hand after defecation? |  |  |
|  | Yes |  |  |
|  | No |  | If q 31 is no, skip to q 32. |
| 29 | What frequency you washing hand after defecation? |  |  |
|  | Regular |  |  |
|  | Irregular |  |  |
| 30 | Have you been eating of raw vegetable? |  |  |
|  | Yes |  |  |
|  | No |  |  |
| 31 | have you regularly been wearing shoes? |  |  |
|  | yes |  |  |
|  | Sometimes |  |  |
|  | Never |  |  |
|  | I don't know |  |  |
| **606** | **STOOL SPECIMEN TEST RESULTS** |  |  |
| 32 | Stool sample results |  |  |
|  | Positive |  |  |
|  | Negative |  |  |
|  | **Checked by supervisor:**  **Name**--------------------------**Signature** --------------------------  Thank you for participating in this interview |  |  |
